# The GRADE evidence-to-decision framework: a report of its testing and application in 15 international guideline panels

**DOI:** 10.1186/s13012-016-0462-y

**Published:** 2016-07-15

**Authors:** Ignacio Neumann, Romina Brignardello-Petersen, Wojtek Wiercioch, Alonso Carrasco-Labra, Carlos Cuello, Elie Akl, Reem A. Mustafa, Waleed Al-Hazzani, Itziar Etxeandia-Ikobaltzeta, Maria Ximena Rojas, Maicon Falavigna, Nancy Santesso, Jan Brozek, Alfonso Iorio, Pablo Alonso-Coello, Holger J. Schünemann

**Affiliations:** 1 Department of Clinical Epidemiology and Biostatistics, McMaster University, Hamilton, Canada; 2 Department of Internal Medicine, School of Medicine, Pontificia Universidad Catolica de Chile, Santiago, Chile; 3 Evidence-Based Dentistry Unit, Faculty of Dentistry, University of Chile, Santiago, Chile; 4 Department of Internal Medicine, American University of Beirut, Beirut, Lebanon; 5 Department of Medicine/Nephrology and Biomedical and Health Informatics, University of Missouri-Kansas City, Kansas City, Missouri USA; 6 Department of Clinical Epidemiology & Biostatistics, McMaster University Health Sciences Centre, Room 2C16, 1280 Main Street West, Hamilton, ON L8S 4K1 Canada; 7 Basque Office for Health Technology Assessment-OSTEBA—Directorate for Health Research and Innovation, Ministry for Health Basque Government, Vitoria-Gasteiz, Spain; 8 Department of Clinical Epidemiology and Biostatistics, Faculty of Medicine, Pontificia Universidad Javeriana, Bogotá, Colombia; 9 Institute for Education and Research, Hospital Moinhos de Vento, Porto Alegre, Brazil; 10 Iberoamerican Cochrane Centre, Biomedical Research Institute Sant Pau (IIB Sant Pau-CIBERESP), Barcelona, Spain

**Keywords:** Clinical practice guidelines, GRADE, Evidence to decisions framework, GRADEpro, Recommendations

## Abstract

**Background:**

Judgments underlying guideline recommendations are seldom recorded and presented in a systematic fashion. The GRADE Evidence-to-Decision Framework (EtD) offers a transparent way to record and report guideline developers’ judgments. In this paper, we report the experiences with the EtD frameworks in 15 real guideline panels.

**Methods:**

Following the guideline panel meetings, we asked methodologists participating in the panel to provide feedback regarding the EtD framework. They were instructed to consider their own experience and the feedback collected from the rest of the panel. Two investigators independently summarized the responses and jointly interpreted the data using pre-specified domains as coding system. We asked methodologists to review the results and provide further input to improve the structure of the EtDs iteratively.

**Results:**

The EtD framework was well received, and the comments were generally positive. Methodologists felt that in a real guideline panel, the EtD framework helps structuring a complex process through relatively simple steps in an explicit and transparent way. However, some sections (e.g., “values and preferences” and “balance between benefits and harms”) required further development and clarification that were considered in the current version of the EtD framework.

**Conclusions:**

The use of an EtD framework in guideline development offers a structured and explicit way to record and report the judgments and discussion of guideline panels during the formulation of recommendations. In addition, it facilitates the formulation of recommendations, assessment of their strength, and identifying gaps in research.

**Electronic supplementary material:**

The online version of this article (doi:10.1186/s13012-016-0462-y) contains supplementary material, which is available to authorized users.

## Background

Clinical practice guidelines are an efficient way to bridge the gap between research evidence, expert experience, and decision-making [1–4]. In order to be trustworthy, guidelines need to be explicit regarding the methods used to summarize the evidence and rate its certainty (also known as quality of the evidence or confidence in the effect estimates) and about the judgments involved in moving from evidence to recommendations [5–7]. Unfortunately, such judgments are rarely recorded and presented in a systematic way.

The Grading of Recommendations Assessment, Development and Evaluation (GRADE) working group (www.gradeworkinggroup.org), a collaborative of over 500 scientists, clinicians, and people with other backgrounds, has developed an approach to assessing the certainty in the body of evidence summarized in systematic reviews that support a decision or guideline recommendation, called the GRADE approach [8, 9]. This approach is used by over 90 organizations, including the World Health Organization, the National Institutes of Health and Care Excellence, the Canadian Task Force for the Preventive Services, numerous professional organizations, and the Cochrane Collaboration. There are over 20,000 citations to GRADE’s methodological work, and thousands of recommendations have been developed following the GRADE approach. GRADE can be used to evaluate different types of evidence, including evidence for intervention effects (including multiple treatment comparisons), test accuracy, prognosis, and resources.

In the context of its “Developing and Evaluating Communication Strategies to Support Informed Decisions and Practice Based on Evidence (DECIDE)” project, GRADE has developed strategies for the targeted communication of evidence-based recommendations to different stakeholders [10]. One of such strategies is the GRADE Evidence to Decision (EtD) Framework, meant to structure development of both recommendations (e.g., clinical recommendations) and decisions (e.g., coverage or public health decisions) using the GRADE approach. Each EtD framework includes detailed sections describing (a) the question and background, (b) an assessment of the evidence, and (c) the conclusions. A summary of the effects of alternative management strategies on patient-important outcomes, data about patients’ values and preferences, and information about resource utilization are part of the assessment section [11–13]. The EtD framework also features information regarding acceptability and feasibility of the alternative management strategies, as well their impact on health equity. This data are obtained from current systematic reviews when available or, more often, updating of previous reviews or the de novo systematic reviews. Finally, The EtD framework provides an explicit record of the judgments made by guideline panelists that ultimately determine the direction and strength of recommendations or decisions. In this paper, we report on the first experience with the EtD framework for clinical recommendations in real guideline panels.

## Methods

### The EtD frameworks

The EtD frameworks evaluated in this study included different versions at various stages of its full development during the DECIDE project. The earliest version of the full GRADE EtD framework was used in 2012 in World Health Organization (WHO) Guidelines for the treatment of multidrug-resistant tuberculosis [14]. The last version of the EtD applied in this study was used in a series of guidelines for the Ministry of Health in Saudi Arabia in 2014 (http://www.moh.gov.sa/depts/Proofs/Pages/Guidelines.aspx). The EtDs were developed based on GRADE evidence to decision tables first utilized in a WHO guideline on avian influenza [15, 16]. The original evidence to decision table (rather than framework) only included five decision criteria [16, 17]. Table 1 describes the iterative changes that were made to the EtD framework during this project. Additional file 1: Table S1 offers an example of the later EtD frameworks tested in this study with 11 criteria and the question and conclusion section [18]. Four panels utilized on-line versions of the interactive EtD frameworks through GRADE’s software GRADEpro [19] while the other panels used paper-based versions (Table 2). All EtDs were transferred for final agreement with panel members and publication of the guideline to GRADEpro.

**Table 1:** Evolution of the EtD framework

Earlier version	Latest version
Used on the World Health Organization and Spanish Guidelines program	Used in Kingdom of Saudi Arabia, World Allergy Organization, Colombian guideline program and rare diseases guidelines
Did not include “prioritization of the problem”	Included a question evaluating the priority of the problem
Included a single question evaluating the balance between health benefits and harms of the intervention of interest	The original question was disaggregated into 3 questions: (1) the magnitude of the health benefits, (2) the magnitude of the health harms, and (3) the balance between health benefits and harms.
Included a single question evaluating the variability of patients’ values and preferences	A judgment regarding the uncertainty about patients’ values and preferences was added.
Included a single question evaluating the cost of the intervention in relation to the benefits	The original question was disaggregated into 2 questions: (1) what is the cost of the intervention and (2) how large is this cost in relation to the benefits.
Included equity as an additional criterion	Acceptability and feasibility were added to equity

**Table 2:** Guideline panels on which the GRADE Evidence to Decision (EtD) Framework was tested

Guideline panels
Kingdom of Saudi Arabia Guideline Program^a^
1. Management of allergic rhinitis in adults and children
2. Antithrombotic Treatment of Patients with Non-valvular Atrial Fibrillation
3. Use of Screening Strategies for Detection of Breast Cancer (used interactive EtD in GRADEpro during panel meeting)
4. Screening and Treatment of Precancerous Lesions for Cervical Cancer Prevention
5. Diagnosis of Suspected First Deep Vein Thrombosis of Lower Extremity
6. Timing of Initiation of Dialysis
7. Role of Vitamin D, Calcium and Exercise in Fracture Prevention in Elderly
8. Use of Thrombolytic Therapy in Acute Stroke
9. Prevention of Venous Thromboembolism in Stroke
10. Treatment of Venous Thromboembolism
World Health Organization Guideline Program^b^
11. The use of bedaquiline in the treatment of multidrug resistant tuberculosis (used interactive EtD in GRADEpro during panel meeting)
WAO Guideline Program^c^
12. Guidelines for allergic diseases prevention (GLAD-P) (used interactive EtD in GRADEpro during panel meeting)
Colombian Guidelines program^d^
13. Clinical practice guidelines for evidence-based care of HIV infection in children
Spanish Guidelines program^e^
14. Spanish Guidelines program: Childhood asthma
Rare disease^f^
15. Hemophilia (used interactive EtD in GRADEpro during panel meeting)

^a^Available at: http://www.moh.gov.sa/endepts/Proofs/Pages/Guidelines.aspx

^b^Available at http://apps.who.int/iris/bitstream/10665/84879/1/9789241505482_eng.pdf?ua=1

^c^Available at: http://www.waojournal.org/content/8/1/4

^d^Available at: http://gpc.minsalud.gov.co/guias/Documents/VIHSida/GPC_corta_VIHpediatrica.pdf

^e^Grupo de trabajo de la Guía de Práctica Clínica sobre asma infantil. Guía de Práctica Clínica sobre asma infantil. Plan de Calidad para el Sistema Nacional de Salud del Ministerio de Sanidad y Política Social. Osteba; 2014. Guías de Práctica Clínica en el SNS: Osteba. Shortly available at: http://portal.guiasalud.es/web/guest/gpc-sns

^f^ https://www.hemophilia.org/sites/default/files/article/documents/NHF-McMaster-Guideline-Care-Models-for-Hemophilia-Management.pdf

We will describe its main features of the EtD here (Additional file 1: Table S1). The header of the table provides details about the clinical question, clearly outlining its components following the PICO framework and any relevant background information. The first column offers the factors or criteria being evaluated framed as questions (e.g., is the problem a priority?). The second column identifies the required judgments by panels and allows recording of these judgments for each criterion. The relevant research evidence and additional considerations upon which the judgments are based redocumented in third column and fourth column, respectively. While the research evidence should be based on systematic reviews of the literature, additional considerations allow capturing specific considerations and information about the local context in which the recommendation will be applied. Finally, the framework captures the judgments leading to the direction and strength of the recommendation or decision. In addition, there is free space to include subgroup and implementation considerations, as well as information regarding futures updates, monitoring, and research priorities. In the four guidelines that used GRADEpro, GRADE’s online tool to assess evidence and develop recommendations, the information was shown on a large screen during the panel meeting and judgments and additional considerations were added online to the EtD during the panel meetings [19]. The version of the EtD in GRADEpro corresponded to the stage of the EtD development (shown in Additional file 1: Table S1) while the current version of GRADEpro that is available on the web (www.gradepro.org) includes the fully developed static and interactive EtD.

### Sample

We used the EtD frameworks in 15 international guideline panels (Table 2). Two guideline panels were organized by international agencies (World Health Organization and World Allergy Organization); 12 panels were in the context of national guideline programs (10 in the Kingdom of Saudi Arabia, 1 in Colombia, and 1 in Spain); and 1 panel was part of an effort to produce establish methods for developing guidance on rare diseases (Italy and Germany).

### Data collection

All guidelines were developed between 2012 and 2014. The guidelines for Saudi Arabia were produced by ten panels, five of them meeting in parallel followed by consecutive meetings of the other five panels. While the preparatory work for these panels was extensive and preceded the panel meetings, the panel meetings lasted 2 days each [20]. Two or three methodologists supervised two panels each sequentially over a total of 4 days. The other guideline panels met over 1 or 2 days and were also led by pairs of methodologists. Following the guideline panel meetings, we asked the methodologists leading the panel to provide feedback regarding the EtD framework. Specifically, three investigators (WW, PAC, HJS) developed a spreadsheet with pre-specified domains and we asked the methodologists to provide free text comments on each domain and also free text comments about their overall experience with the framework (Additional file 2). Two of the three investigators (WW and HJS) who developed the data collection sheet also participated as methodologists in guideline panels and therefore provided comments and feedback to the EtD framework. We choose this method for collecting the data given its relatively quick implementation just after the panel meetings. We instructed the methodologists to consider their own experience with the EtD framework and also the feedback collected from the rest of the panelists during the guideline panel meeting. After a first round of feedback, we provided a summary of the results to the methodologists for further input.

**Table 3:** Summary of the results

Section in the EtD table	Compiled feedback
Order of columns and rows	The order was not a relevant issue for most panels
Criteria in EtD	One methodologist expressed concerns regarding the overlap between the sections “acceptability” and “feasibility” with “values and preferences” and “resource considerations”. Another methodologist considered the section “priority” redundant. According to another methodologist, the criteria of the table were not easy to apply to recommendations addressing multiple comparisons.
Judgment column	Two methodologists stated that allowing modifications to the answer options might be needed to accommodate different contexts and scenarios.
Research evidence	Although for the majority of panels there were no issues with this column, one methodologist stated that the difference between the purposes of the columns “available evidence” and “additional consideration” was not clear.
Additional considerations	One methodologist suggested using this column to summarize the available evidence as a general narrative statement.
Background	One methodologist considered this section redundant (with respect to information that is present in the main text of the guideline).
PICO	Three methodologists expressed concerns regarding how the question is presented, specifically, they suggested making the PICO structure more explicit (and using the exact terms that P-I-C-O stand for).
Perspective	One methodologist considered this section particularly relevant, as being explicit about the perspective may help to make transparent the decisions made to formulate recommendations.
Overall certainty of the Evidence	In general, this section was well evaluated by methodologists, with no major suggestion for improvement. Two methodologists made minor wording suggestions. Another methodologist suggested expanding the content to include more information about each particular outcome.
Values and preferences (“Uncertainty about how much people value the main outcomes”)	This section posed significant difficulties in several panels. Four methodologists suggested differentiating between “variability” and “uncertainty” of patients’ values and preferences. Another methodologist suggested making explicit the source of patients’ values and preferences. The order of presenting and discussing values and preferences was also questioned and subsequently changed in the EtD framework.
Balance of benefits and harms	There were major difficulties in 6 guideline panels. According to methodologists, panelists had problems answering consistently the questions about the size of the effect. Additionally, two methodologists considered the questions of this section redundant.
Resource use	Three guideline panels (all without health economists) struggled answering the question about the relationship between incremental cost and benefits. These panels proposed “is the treatment cost-effective?” as a better alternative. The only guideline panel with health economists considered the questions of this section too superficial.
Equity	Three guideline panels struggled with this question. A more clear definition of health equity and more guidance on how to answer the question were considered necessary. Two methodologists suggested adding the option “no effect on health equity” to the answers options.
Acceptability	Two methodologists expressed problems when trying to identify the relevant stakeholders. More guidance was considered necessary.
Feasibility	None
Panel decisions	One methodologist considered the wording of this section confusing when the recommendation under discussion involved two active treatments. Another methodologist suggested de-emphasizing the option “no recommendation”
Justification/remarks	Two panels struggled to decide what to include in the remarks. More guidance was considered necessary.
Subgroup considerations	One methodologist considered that subgroups should be more explicit in the table.
Implementation considerations	According to methodologists, this section was used in different ways across guideline panels. More guidance in what to include was considered necessary.
Monitoring and evaluation	None
Research priorities	In general, there was agreement regarding the importance of this section.

### Data analysis

Two investigators (IN and RBP) independently identified themes from the feedback obtained from the methodologists. They used the pre-specified domains as coding system. Then, the two investigators jointly interpreted the data and organized the themes into the following categories: general comments about the framework and comments about specific sections within the framework. All participants were invited to review and comment on a draft report of the results.

## Results

### Context and methodologists

The topics covered by guideline panels were diverse, including cardiovascular diseases (5 guidelines), asthma and allergy (3 guidelines), infectious diseases (2 guidelines), cancer screening and diagnosis (2 guidelines), and others (3 guidelines). Most of the guidelines were focused on adults (11 guidelines) (Table 2).

Ten methodologists led the guideline development on the 15 panels, and for all guidelines, a second methodologist provided backup support. Seven of the panel leads were physicians, two dentists, and one registered nurse, all of them with postgraduate training in health research methods or a related discipline. All guideline methodologists had formal training and experience in conducting systematic reviews and in using the GRADE approach to formulate recommendations. They were all members of the GRADE working group. For 13 out of the 15 guidelines, one highly experienced guideline methodologist led the panel discussions supported by another guideline methodologist. Most of the highly experienced methodologists had led multiple systematic reviews and high level guideline panels at major international organizations (e.g., WHO) or professional societies with large guideline programs (e.g., American Thoracic Society or American College of Chest Physicians). The gender distribution was six males and four females, and the mean age was 37.8 years (standard deviation 6.3). All of them provided feedback about the EtD framework.

### General comments about the EtD framework

Table 3 summarizes the results by themes. In general, the EtD framework was well received and the comments were mostly positive. The methodologists felt that in a real guideline panel, the EtD framework helps structuring a complex group process through relatively simple steps in an explicit and transparent way. Further, two methodologists commented that the explicitness of the framework could help having an accurate idea of the underlying evidence and protecting against inappropriate recommendations.

Two methodologists stated that the wording of the questions and options to answer were suboptimal for recommendations comparing two active interventions (instead of one active intervention against placebo/no treatment). Also, three methodologists raised concerns regarding the length of the framework and the relevance of some sections in specific circumstances. In particular, considerations regarding feasibility, accessibility, and equity were considered less relevant in the context of clinical recommendations. Finally, two methodologists stated that in order to take full advantage of the ETD framework, it is necessary to be familiar with the GRADE approach and with the framework itself. Therefore, for new users, previous training may be helpful for successful implementation and use. For example, one methodologist stated, “Until you see the process you don’t appreciate all the real work and value of the forms and filling out information before the panel meeting, etc. You must go through the process and use the [EtD] and see the outcome.”

### Comments about specific sections

The sections “values and preferences” and “balance of benefits and harms” posed difficulties in several panels. Regarding the “values and preferences” section, the GRADE approach requires guideline panelists to judge whether there is important variability in how patients’ value the main outcomes and to what extent there is important uncertainty about this. In case of any of the two, panels should consider issuing a weak recommendation. The main concern with this question was that the answer options did not differentiate between the variability and uncertainty in patients’ values and preferences, creating some confusion among panelists. Four methodologists suggested dividing this question in two.

Regarding the balance between benefits and harms, this section features three questions requiring panelists to judge the magnitude of desirable and undesirable effects, as well the relation between the two. Panelists struggled judging the size of the benefits and harms consistently. Two methodologists considered the three questions redundant and suggested to drop the questions about the size of the benefit and harms and maintain only the one addressing their balance.

## Discussion

This is the first study evaluating the use of the GRADE EtD framework in the context of real guideline development. Given the widespread application of GRADE, this study provides important information and rationale for utilizing EtDs in guidelines. In general, the response to the use of the EtD framework was positive, although some sections (i.e., “values and preferences” and “balance between benefits and harms”) required improvements, in particular the choice of answer options. These improvements have been implemented in the current final option of GRADE EtD frameworks (www.gradepro.org) [11–13].

There are some limitations to this study. The use of iteratively developed formats of the EtD framework in different guideline panels could have introduced some variability in the provided answers. However, it was necessary as part of the development and improvement of the EtDs. For example, early testing with the World Health Organization and World Allergy Organization panels revealed difficulties with providing responses to the available answer options. In order to make best use of the EtDs, they were modified for use in the work of later guideline panels. While methodologists were prospectively instructed before the panel meetings to record challenges with the EtDs during the panel meetings, there was no independent evaluation of challenges with the EtD frameworks. However, the data were recorded without delay and we believe that recall of the encountered issues does not lead to bias as all methodologists had an interest in improving the frameworks.

The strengths of this study include the strategies taken to ensure the trustworthiness of our findings: prospective data collection, independent and duplicate analysis, and member checking of the conclusions. The most important strength of this work is the application of the EtD frameworks in the context of real guideline development by international panels in a broad range of topics, including diagnostic and therapeutic questions.

Our findings strongly suggest that the EtD framework can enhance the transparency of guideline developers’ judgments, allowing decision makers to truly assess the recommendation. This is not only corroborated by this work but by the adoption of the EtD approach by major organizations such as WHO [21]. What happens within guideline panels is seldom reported in current guidelines [22–26]. Hence, when recommendations are analyzed at a deeper level, some decisions of the panel might seem arbitrary and unjustified. Providing details about the reasons and judgments behind such decisions enrich the recommendations and also may facilitate the adaptation of international guidelines to local contexts. In the EtDs, each criterion is considered by working trough the EtD sequentially and then in a summary before formulating a recommendation. This avoids unnecessarily moving back and forth between criteria and wasting time. In addition, each criterion requires a judgment that determines the direction and strength of a recommendation, which is often not found in guidelines. This information will facilitate updating (by seeing judgments and evaluating if the should change) and adaptation of guidelines (by making others aware of the judgments).

The EtD framework also offers the opportunity to identify and potentially avoid inappropriate recommendations. One potential problem of recommendations is the mismatch between the strength of the recommendation and the underlying information about the certainty in effect estimates (also known as confidence in the evidence or quality of the evidence), the balance between the benefits and harms, values and preferences, and resource considerations [27]. The EtD framework, on the one hand, allows users to judge to what extent the recommendation corresponds to the underlying information and, on the other hand, may help preventing guideline developers from inappropriately grading the strength of the recommendation [28]. Finally, the EtD framework may also be a valuable research tool, since it would allow a detailed comparison of conflicting recommendations from different organizations.

## Conclusions

In conclusion, the use of an EtD framework in guideline development offers an explicit and transparent way to record and report the judgments and discussion of guideline panelists. The EtD framework may facilitate the assessment of recommendations and also their potential adaptation and implementation. As result of this work, a new section was added to the EtD framework. This section allows summarizing the judgments of guideline panelists just prior to the deliberations regarding the direction and strength of recommendations. The currently latest version of the EtD resulting from this and other work including an interactive version of the EtD based on this and other work is available in GRADE’s online GRADEpro Guideline Development Tool (www.gradepro.org) and described elsewhere [11–13]. While the current formats of EtD frameworks are already used widely, future use and research will inform changes that will have to be made to the EtDs. To name some areas of relevance, additional research could identify how to best integrate issues arising from potential conflicts of interest, how to structure research recommendations, how to address agreement of panel members with information provided by systematic reviews, how to document alterations in judgments about the certainty in the evidence by panel members as a result of indirectness of the evidence [29], and how to structure implementation considerations. The GRADE working group will further evaluate and develop the EtD frameworks. Doing this, the evaluation of the EtD frameworks in real guideline development processes should continue to provide information for improvement.

## Additional files

Additional file 1: Table S1. GRADE Evidence to Decision (EtD) Framework example. (DOC 188 kb)

Additional file 2: Example of a EtD framework. (XLSX 21 kb)
