# Supplementary material for: The GRADE evidence-to-decision framework: a report of its testing and application in 15 international guideline panels
Source: Implement Sci. 2016 Jul 15;11:93. doi: 10.1186/s13012-016-0462-y (PMC4946225; doi:10.1186/s13012-016-0462-y)
Supplement: Supplementary file 1 — GRADE Evidence to Decision (EtD) Framework example. (DOC 188 kb) [file 13012_2016_462_MOESM1_ESM.doc]

### Additional file 1: Table S1. GRADE Evidence to Decision (EtD) Framework example

| Question 1: Should intranasal corticosteroids be used in patients with allergic rhinitis (AR)? | |
| --- | --- |
| ***Problem:*** Allergic Rhinitis (seasonal and perennial)  ***Option:*** intranasal corticosteroids  ***Comparison:*** No intranasal corticosteroids  ***Setting:*** Outpatient  ***Perspective:*** Health Care system | ***Background:*** Allergic rhinitis (AR) is defined clinically by nasal hypersensitivity symptoms induced by an immunologically mediated (most often IgE-dependent) inflammation after the exposure of the nasal mucous membranes to an offending allergen. Symptoms of rhinitis include rhinorrhea, nasal obstruction or blockage, nasal itching, sneezing, and postnasal drip that are reversible spontaneously or under treatment. Allergic conjunctivitis often accompanies allergic rhinitis.  Allergic rhinitis has been traditionally subdivided into seasonal, perennial, and occupational rhinitis. Perennial allergic rhinitis is most frequently, although not necessarily, caused by indoor allergens such as house dust mites, moulds, cockroaches, and animal dander. Seasonal allergic rhinitis is most often caused by outdoor allergens such as pollens or moulds. As in a 2010 edition of ARIA guideline in this document we retained the terms “seasonal” and “perennial” to enable the interpretation of published studies, and we also include the terms used to classify AR according to the duration of symptoms as “intermittent” rhinitis (symptoms are present less than 4 days a week or for less than 4 weeks) or “persistent” (symptoms are present at least 4 days a week and for at least 4 weeks).  These guidelines do not address the issues related to diagnosis of allergic rhinitis and it is assumed that the correct diagnosis had been established before commencing treatment. |

|  | [Criteria](#Criteria) | [Judgements](#Judgements) | [research EVIDENCE](#Evidence) | [ADDITIONAL CONSIDERATions](#Information) |
| --- | --- | --- | --- | --- |
| PROBLEM | [Is the problem a priority?](#Priority_E) | | No | Probably  No | Uncertain | Probably  Yes | Yes | [**Varies**](../../../../C:%5CUsers%5Caox%5CAppData%5CLocal%5CMicrosoft%5CWindows%5CTemporary%20Internet%20Files%5CContent.Outlook%5CA5320PQD%5CVaries) | | --- | --- | --- | --- | --- | --- | | |  | | --- | | |  | | --- | | |  | | --- | | |  | | --- | | | x | | --- | | |  | | --- | | | 1. Overall risk of AR in adults Saudi Arabia is 90 per 1000 (79% SAR)  Overall in the Middle East:   - Runny nose, nasal and throat itching, postnasal drip, and nasal congestion or stuffed up nose were the most common and bothersome symptoms of AR. - 58% of participants with AR reported that the condition had an impact on their daily private and professional life. - 72% reported that limitations on their work/school activities - 35% reported that interfered with and caused them to miss work or - Sleep disturbances were shown in this survey to be extremely troubling in 15% of AR patients.   (Abdulrahman H, 2012. Survey conducted in Middle East including KSA)  2. A high percentage of patients with AR surveyed missed work or had their work performance affected by allergies: work productivity decreasing by 23% in AIA, 24% in AIAP, 33% in AILA and 30% in Middle East when allergy symptoms were at their worst.  Nasal allergies also interfered with many patients' sleep, and were associated with feelings of depression, anxiety, irritability and tiredness.  (Blaiss 2012, America, Asia pacific, Latin America, and Middle East) | The guideline panel estimates a prevalence of 20% to 40% of AR in KSA. They consider that due to the lack of an appropiate data base with these data, the self- reporting studies could underestimate the prevalence (for not recognize the symptoms or not having a medical diagnosis) or overestimate (for considering any kind of rhinitis not only the allergic one). |

**Seasonal / Intermittent Allergic Rhinitis**

|  | [Criteria](#Criteria) | [Judgements](#Judgements) | [research EVIDENCE](#Evidence) | [ADDITIONAL considerations](#Information) |
| --- | --- | --- | --- | --- |
| BENEFITS & HARMS OF THE OPTIONS | [What is the overall certainty of this evidence?](#Overall_certainty_C) | | No included studies | Very low | Low | Moderate | High | | --- | --- | --- | --- | --- | | |  | | --- | | |  | | --- | | |  | | --- | | | x | | --- | | |  | | --- | | | | Outcome | Relative importance | Certainty of the evidence (seasonal AR) | | --- | --- | --- | | Nasal symptoms | Critical | Moderate | | Nasal congestion | Critical | Moderate | | Rhinorrhea | Critical | Moderate | | Sneezing | Important | Moderate | | Nasal itching | Important | Moderate | | Ocular symptoms | Important | Moderate | | Quality of life | Critical | Moderate | | Adverse effects | Critical | Moderate |   Summary of the evidence for patients’ values and preferences: See aditional considerations columm.  High value on the moderate effect of intranasal glucocorticosteroids reducing symptoms, and a relatively low value on avoiding their possible moderate adverse effects.  Summary of findings: See evidence table and reference list  1. Relative importance of AR symptoms(Revicki 1998 (US), Lo 2006 (China))  Rhinitis Symptom Utility Index (RSUI):  0 –best state of symptoms-no symptoms. 1 – the worst state symptoms- 8-14 days with severity symptoms.  The mean RSUI score for this sample was 0.72 ± 0.23, with a range of 0.15–1.0. (Revicki 1998 (US), Lo 2006 (China))  2. In the treatment of nasal allergies worldwide. The allergy surveys highlight the key factors in choosing an INCS: fast, complete, and long-lasting symptom relief. Furthermore, Comparing with the results of others allergy surveys worldwide a higher proportion of patients in the Middle East reported bothersome side effects of their prescription nasal sprays, and a higher proportion of these patients strongly agreed that there were no truly effective treatments for allergic rhinitis. This suggests that health care practitioners in the Middle East should be encouraged to explain the use of INCSs in greater depth to their patients. Patient education must play a central role in treatment decision making, particularly in the Middle East, to achieve higher patient satisfaction. (Hadi, U, 2013. WordWide including KSA).  3. The most common reasons cited for dissatisfaction with INCS medications were inadequate effectiveness, bothersome side effects (e.g., unpleasant taste and retrograde drainage into the pharynx), decreased effectiveness with chronic use, and failure to provide 24-hour relief. (Abdulrahman H, 2012. Middle East including KSA).  4. Narrative satisfaction and preference for INCS: Only 19% stated the INCSs as being effective/important drugs, while 36% stated them as being dangerous drugs. In reply to the question “would you use nasal steroids if they were prescribed?”, 47% of the entire study sample answered “yes, if prescribed”. (Cingi 2010, Turkey)  5. Narrative satisfaction and preference for treatment: Nasal sprays were not used daily because their use was inconvenient and embarrassing. Factors such as mild disease, side-effects, cost, and lack of efficacy were of less importance. (Borres 1997, Sweden) |  |
|  | [Is there important uncertainty about how much people value the main outcomes?](#Values_E) | \| Important uncertainty or variability \| Possibly important uncertainty or variability \| Probably no important uncertainty or variability \| No important uncertainty or variability \| No known undesirable outcomes \| \| --- \| --- \| --- \| --- \| --- \| \| \|  \| \| --- \| \| \|  \| \| --- \| \| \|  \| \| --- \| \| \| x \| \| --- \| \| \|  \| \| --- \| \| |  |  |
|  | [Are the desirable anticipated effects large?](#Desirable_C) | \| No \| Probably  No \| Uncertain \| Probably  Yes \| Yes \| [**Varies**](../../../../C:%5CUsers%5Caox%5CAppData%5CLocal%5CMicrosoft%5CWindows%5CTemporary%20Internet%20Files%5CContent.Outlook%5CA5320PQD%5CVaries) \| \| --- \| --- \| --- \| --- \| --- \| --- \| \| \|  \| \| --- \| \| \|  \| \| --- \| \| \|  \| \| --- \| \| \| x \| \| --- \| \| \|  \| \| --- \| \| \|  \| \| --- \| \| |  |  |
|  | [Are the undesirable anticipated effects small?](#Undesirable_C) | \| No \| Probably  No \| Uncertain \| Probably  Yes \| Yes \| [**Varies**](../../../../C:%5CUsers%5Caox%5CAppData%5CLocal%5CMicrosoft%5CWindows%5CTemporary%20Internet%20Files%5CContent.Outlook%5CA5320PQD%5CVaries) \| \| --- \| --- \| --- \| --- \| --- \| --- \| \| \|  \| \| --- \| \| \|  \| \| --- \| \| \|  \| \| --- \| \| \| x \| \| --- \| \| \|  \| \| --- \| \| \|  \| \| --- \| \| |  |  |
|  | [Are the desirable effects large relative to undesirable effects?](#Values_E) | \| No \| Probably  No \| Uncertain \| Probably  Yes \| Yes \| [**Varies**](../../../../C:%5CUsers%5Caox%5CAppData%5CLocal%5CMicrosoft%5CWindows%5CTemporary%20Internet%20Files%5CContent.Outlook%5CA5320PQD%5CVaries) \| \| --- \| --- \| --- \| --- \| --- \| --- \| \| \|  \| \| --- \| \| \|  \| \| --- \| \| \|  \| \| --- \| \| \|  \| \| --- \| \| \| x \| \| --- \| \| \|  \| \| --- \| \| |  |  |

|  | [Criteria](#Criteria) | [Judgements](#Judgements) | [research EVIDENCE](#Evidence) | [ADDITIONAL considerAtions](#Information) | | |
| --- | --- | --- | --- | --- | --- | --- |
| RESOURCE USE | [Are the resources required small?](#Costs_C) | | No | Probably  No | Uncertain | Probably  Yes | Yes | [**Varies**](../../../../C:%5CUsers%5Caox%5CAppData%5CLocal%5CMicrosoft%5CWindows%5CTemporary%20Internet%20Files%5CContent.Outlook%5CA5320PQD%5CVaries) | | --- | --- | --- | --- | --- | --- | | |  | | --- | | |  | | --- | | |  | | --- | | | x | | --- | | |  | | --- | | |  | | --- | | | - The average treatment cost per patient in Canada over 12 months in fluticasone Intranasal was CAD 508.06 (Ståhl 2000, Canada), with a drug cost per patient of 214 CAD, which was an average around 120 CAD more expensive than the cost of budesonide intranasal. | | | **- Average annual cost per patient: around 600 SAR**  Average price of 120 doses Spray (a month treatment): 43 SAR. |
|  | [Is the incremental cost small relative to the net benefits?](#Cost_effectiveness_C) | \| No \| Probably  No \| Uncertain \| Probably  Yes \| Yes \| [**Varies**](../../../../C:%5CUsers%5Caox%5CAppData%5CLocal%5CMicrosoft%5CWindows%5CTemporary%20Internet%20Files%5CContent.Outlook%5CA5320PQD%5CVaries) \| \| --- \| --- \| --- \| --- \| --- \| --- \| \| \|  \| \| --- \| \| \|  \| \| --- \| \| \|  \| \| --- \| \| \| x \| \| --- \| \| \|  \| \| --- \| \| \|  \| \| --- \| \| | None identified | |  | |
| EQUITY | [**What would be the impact**](#Equity_C)  [on health inequities?](#Equity_C) | | Increased | Probably  increased | Uncertain | Probably  reduced | Reduced | [**Varies**](../../../../C:%5CUsers%5Caox%5CAppData%5CLocal%5CMicrosoft%5CWindows%5CTemporary%20Internet%20Files%5CContent.Outlook%5CA5320PQD%5CVaries) | | --- | --- | --- | --- | --- | --- | | |  | | --- | | |  | | --- | | |  | | --- | | | x | | --- | | |  | | --- | | |  | | --- | | | None identified | |  | |
| ACCEPTABILITY | [**Is the option acceptable  to key stakeholders?**](#Acceptability_C) | | No | Probably  No | Uncertain | Probably  Yes | Yes | [**Varies**](../../../../C:%5CUsers%5Caox%5CAppData%5CLocal%5CMicrosoft%5CWindows%5CTemporary%20Internet%20Files%5CContent.Outlook%5CA5320PQD%5CVaries) | | --- | --- | --- | --- | --- | --- | | |  | | --- | | |  | | --- | | |  | | --- | | |  | | --- | | | x | | --- | | |  | | --- | | | None identified | |  | |
| FEASIBILITY | [**Is the option feasible to implement?**](#Feasibility_C) | | No | Probably  No | Uncertain | Probably  Yes | Yes | [**Varies**](../../../../C:%5CUsers%5Caox%5CAppData%5CLocal%5CMicrosoft%5CWindows%5CTemporary%20Internet%20Files%5CContent.Outlook%5CA5320PQD%5CVaries) | | --- | --- | --- | --- | --- | --- | | |  | | --- | | |  | | --- | | |  | | --- | | |  | | --- | | | x | | --- | | |  | | --- | | | None identified | |  | |

| [Balance of consequences](#Consequences_C) | Undesirable consequences  *clearly outweigh*  desirable consequences in most settings | Undesirable consequences *probably outweigh*  desirable consequences in most settings | The balance between  desirable and undesirable consequences  *is closely balanced or uncertain* | Desirable consequences  *probably outweigh* undesirable consequences in most settings | Desirable consequences  *clearly outweigh*  undesirable consequences in most settings |
| --- | --- | --- | --- | --- | --- |
|  | |  | | --- | | |  | | --- | | |  | | --- | | |  | | --- | | | x | | --- | |

| [Type of recommendation](#TypeofRecommendation_C) | We recommend against  offering this option | We suggest not offering  this option | We suggest offering  this option | We recommend offering  this option |
| --- | --- | --- | --- | --- |
|  | \|  \| \| --- \| | \|  \| \| --- \| | \|  \| \| --- \| | \| x \| \| --- \| |
| [Recommendation (text)](#Recommendation_C) | The KSA MoH panel recommends Intranasal corticosteroids for treatment of adults with seasonal or intermittent allergic rhinitis (Strong recommendation; Moderate-quality evidence). | | | |
| [Justification](#Justification_C) | The evidence, with an overall moderate certainty, shows that the desirable effects probably are large relative to undesirable effects. It is considered that there is no important uncertainty or variability about how much people value its effectiveness and its mild adverse effects. The incremental cost is probably small relative to the net benefits due to relatively low cost of the drugs. Furthermore, the use of INSC would be acceptable and feasible. Reasons to formulate a strong rather than a conditional recommendation. | | | |
| [Subgroup considerations](#Justification_C) | - Health care practitioners in the Middle East should be encouraged to explain the use of INCSs in greater depth to their patients especially about the time required to reach the desired symptom relief. | | | |
| [Implementation considerations](#Implementation_C) | - Different INCS should be available to provide choice opportunity for different patient preferences related with drug characteristics, such as smell for example. | | | |
| [Monitoring and evaluation](#Monitoring) | None described. | | | |
| [Research priorities](#Research) | Nation-wide population-based community prevalence studies are needed to correctly estimate the AR rates. Patient values and preferences and cost effectiveness studies are also needed in the context of KSA to inform future guidelines and stakeholders.  Further research is needed to answer the question about the efficacy and specially safety of intranasal glucocorticosteroids in children with AR.  A complete rigorously performed and reported systematic review of all individual intranasal glucocorticosteroids (budesonide, ciclesonide and beclomethasone) versus placebo that provides information on all outcomes important to patients, including adverse effects, is required. | | | |
